# Pyrosequencing quantified methylation level of miR-124 predicts shorter survival for patients with myelodysplastic syndrome

**DOI:** 10.1186/s13148-017-0388-5

**Published:** 2017-08-30

**Authors:** Hong Wang, Tong-Tong Zhang, Song Jin, Hong Liu, Xiang Zhang, Chang-Geng Ruan, De-Pei Wu, Yue Han, Xiao-Qin Wang

**Affiliations:** 1 Jiangsu Institute of Haematology, Institute of Blood and Marrow Transplantation, Department of Haematology, Collaborative Innovation of Haematology, Key Laboratory of Thrombosis and Hemostasis of Ministry of Health, The First Affiliated Hospital of Soochow University, 188 Shi Zi Street, Suzhou, 215000 China; 2 Department of Haematology, Huashan Hospital of Fudan University, 12 Wulumuqi Road Central, Shanghai, 200040 China

**Keywords:** miR-124, Methylation, MDS, Prognosis, Pyrosequencing

## Abstract

**Background:**

Aberrant CpG island methylation has been increasingly recognized as a common event in myelodysplastic syndrome (MDS). To date, most of the previous studies of miR-124 in MDS have focused on epigenetic changes and little is known about the underlying mechanism through which miR-124 regulates *CDK6* expression.

**Results:**

In the present study, we employed pyrosequencing analysis to quantify the methylation levels of upstream regions of the miR-124 genes (miR-124-1, miR-124-2 and miR-124-3) in 56 primary MDS patients. We found the three miR-124 genes were methylated in MDS patients. Univariate analysis revealed that the World Health Organization (WHO) classification, marrow blast count, karyotype, International Prognostic Scoring System (IPSS), mean corpuscular volume, as well as high methylation of miR-124-1, miR-124-2 and miR-124-3 were significantly related to overall survival. In leukaemia-free survival, patients who were older and had an advanced WHO classification, high marrow blast counts, high IPSS risk and high methylation of miR-124-1 and miR-124-2 progressed rapidly to acute myeloid leukaemia. Multivariate analysis demonstrated that high methylation of miR-124-3 was an independent factor of overall survival. Median survival of patients with high miR-124-3 methylation was significantly shorter (7.6 months) than patients with low methylation (32.7 months; *P* = 0.010). A functional study revealed that silencing of miR-124 resulted in upregulation of its target gene, cyclin dependent kinase *CDK6*, which in turn promoted cell proliferation in the MDS cell line SKM-1.

**Conclusions:**

High methylation of miR-124-3 predicts shorter survival for patients with MDS, which may be a useful prognostic marker in MDS.

**Electronic supplementary material:**

The online version of this article (doi:10.1186/s13148-017-0388-5) contains supplementary material, which is available to authorized users.

## Background

Myelodysplastic syndrome (MDS) is a clonal disorder characterized by dysplastic and ineffective haematopoiesis and a high risk of acute myeloid leukaemia (AML) progression. In recent years, aberrant CpG island methylation has been increasingly recognized as a common event in MDS, and the relation between prognosis and DNA methylation has been demonstrated [1–4].

MicroRNAs (miRNAs) are a group of small non-coding RNAs consisting of 18–22 nucleotides that negatively regulate mRNA transcripts, typically by base pairing with a complementary region in the 3′-untranslated region (3′-UTR) of the target gene [5]. They play pivotal roles in a wide range of biological processes including proliferation, apoptosis and differentiation [6]. Increasing evidence indicates that miRNAs are involved in carcinogenesis, either oncogenically, when tumour suppressor genes are targeted, or by tumour suppression, when oncogenes are targeted [7]. Dysregulation of miRNAs has been found to be associated with the clinical outcomes of haematological malignancies, such as miR-212, miR-3151, miR-181 and miR-29b in AML [8–11], miR-26A1 in chronic lymphocytic leukaemia [12], and miRNA-194-5p in MDS [13].

Human miR-124 is encoded at three loci: miR-124-1 (8p23.1), miR-124-2 (8q12.3) and miR-124-3 (20q13.33). Downregulation of miR-124 by promoter methylation has been observed in various types of cancer, including gastric, colon, prostate, cervical and pancreatic cancers [14–18]. In haematological malignancies, Lujambio et al. first reported the epigenetic silencing of miR-124 in acute lymphoblastic leukaemia (ALL) [19]. Subsequently, inhibited expression of miR-124 due to abnormal methylation was demonstrated in AML and MDS [20–22]. In ALL, miR-124 methylation was shown to be an independent prognostic factor in predicting overall survival and disease-free survival [23]. Previous research confirmed that miR-124 was epigenetically silenced in various types of cancer and regulated cell biological behaviours by targeting several important genes, such as *STAT3*, *ROCK1*, *EZH2*, *RAC1* and *CD164* [18, 24–27]. Recent evidence has shown that miR-124 inhibited cell proliferation by directly targeting the cyclin dependent kinase *CDK6* in ALL and myeloma [19, 23, 28]. In MDS, Castoro et al. found that patients with miR-124 re-expression who responded to DNA methylation inhibitor decitabine (DAC) had a significant decrease of *CDK6* expression [21]. To date, most of the previous studies of miR-124 in MDS have focused on epigenetic changes and little is known about the underlying mechanism through which miR-124 regulates CDK6 expression.

In the present study, we used pyrosequencing analysis to precisely quantify the methylation levels of upstream regions of the miR-124 genes (miR-124-1, miR-124-2 and miR-124-3) in 56 primary MDS patients to determine whether they are methylated in this disorder. We also analysed the correlation between different methylation levels and clinical features to evaluate their prognostic significance in MDS patients. To further clarify the underlying mechanism through which miR-124 regulates its target, CDK6, we performed a functional study in the SKM-1 cell line in vitro.

## Methods

### Patients and SKM-1 cell line

Bone marrow samples were obtained from 56 adult patients with primary MDS who were diagnosed at four hospitals in Shanghai, China between June 2003 and April 2007. All samples were collected after informed consent had been obtained and in accordance with the institutional guidelines of the Shanghai Leukaemia Cooperative Group and with the Helsinki Declaration. The study was approved by the Committees for the Ethical Review of Research at Shanghai Leukaemia Cooperative Group. Diagnosis was made according to the 2016 WHO classification [29]. The patients comprised 36 men and 20 women with a median age of 65 years (range 20–84 years). According to the 2016 WHO classification, there were 5 patients with single lineage dysplasia (SLD), 7 with multilineage dysplasia (MLD), 5 with ring sideroblasts (RS, including 2 with RS-SLD and 3 with RS-MLD), 15 with excess blasts-1 (EB-1), 21 with EB-2, 1 with MDS unclassifiable (MDS-U) and 2 with isolated del(5q-). Cytogenetic examination was carried out on samples from 55 patients. The prognostic score for each patient was calculated using the International Prognostic Scoring System (IPSS) [30]. Patients with lower blast counts (<5%), including SLD, MLD, RS, MDS-U and isolated del(5q-), received supportive care, including blood transfusion and the use of erythropoietin. Patients with EB-1 and EB-2 received AML-like chemotherapy at low doses. All patients were followed until death from any cause or until the last follow-up date (30 April 2008). At the last follow-up date, 34 patients (60.7%) had died and 15 (26.8%) had progressed to AML.

The MDS SKM-1 cell line was derived from a peripheral blood sample of a 76-year-old Japanese male patient at the leukaemia stage who was initially diagnosed as MDS EB-2 [31]. The SKM-1 cell line was purchased from the Health Science Research Resources Bank, Japan. Cells were grown at 37 °C under 5% CO_2_ in RPMI-1640 medium with 10% foetal bovine serum.

### DNA isolation, sodium bisulphite conversion and pyrosequencing analysis

**Fig. 1:**
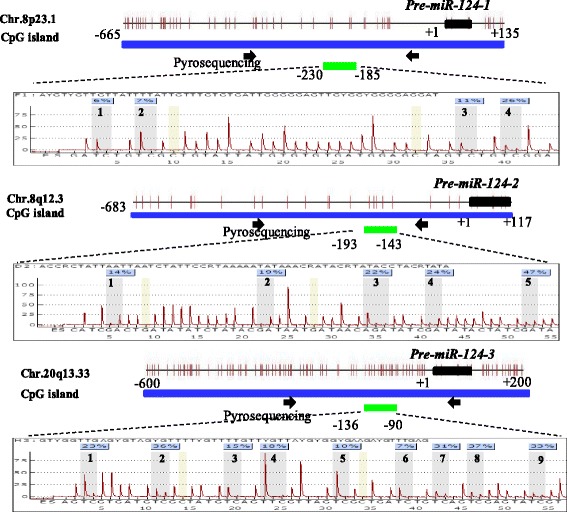
The genomic locations of the bisulfite pyrosequencing assays and the representative results of methylation levels of CpG sites in three miR-124 genomic loci evaluated by pyrosequencing. The positions of the pre-miR-124-1, pre-miR-124-2 and pre-miR-124-3 sequences are indicated by *black* boxes, and the start site is designated as + 1. Each vertical bar represents an individual CpG site, 4 sites was marked in miR-124-1, 5 sites in miR-124-2 and 9 sites in miR-124-3. Primers for PCR amplification are indicated by *black arrows*, and the sequencing location is indicated by a *green* box

Genomic DNA was isolated from the bone marrow samples of 56 MDS patients using a QIAamp DNA Blood Mini Kit according to the manufacturer’s instructions (Qiagen). We also isolated genomic DNA from peripheral blood of 10 healthy donors. Sodium bisulphite modification of the DNA was performed using an EpiTect Bisulfite Kit (Qiagen). MiR-124 was represented at three genomic loci [miR-124-1 (8p23.1), miR-124-2 (8q12.3) and miR-124-3 (20q13.33)]. One CpG-rich region for each of these loci was identified using the CpG Island Searcher, and one pair of primers was designed to analyse the CpG sites. The primer sequences were as follows: miR-124-1: forward: GGGGAGAATAAAGAGTTTTTGGA, reverse: TACTCAACCAACCCC ATTCTTAA; miR-124-2: forward: GTGTGTTGTAAATGGTATGGAGATAT, reverse: CCCAACTCCTATCTCTACTCATCT; and miR-124-3: forward: AAA GGGAGAAGTGTGGGT, reverse: CCCAAAAAAACCCTCAAAACT. PCRs were performed with a PyroMark PCR Kit (Qiagen) under the following conditions: 95 °C for 3 min; 50 cycles of 95 °C for 15 s, 54 °C for 30 s and 72 °C for 30 s; and an elongation step of 72 °C for 5 min. The success of amplification was assessed by 2% agarose gel electrophoresis. The DNA methylation level of the specific CpG nucleotides was then evaluated by pyrosequencing. The sequencing primers were as follows: miR-124-1: GAATAAAGAGTTTTTGGAAG; miR-124-2: TCTCTAA CACATCTACCAAA; and miR-124-3: GGAGGATTGGGATAGTATA. Quantitative pyrosequencing analysis was performed using a PyroMark Q96 ID platform (Qiagen) according to the manufacturer’s protocol. The pyrosequencing assays interrogated 4–9 adjacent CpG sites close to the primary (pri)-miR-124. Output data were analysed using PyroMark Q24 2.0.6 Software (Qiagen). The genomic locations of the bisulphite pyrosequencing assays and the number of investigated CpG sites in each assay are shown in Fig. 1.

### Oligonucleotide transfection

miRNA mimics are small, chemically modified double-stranded RNAs that mimic endogenous miRNAs and enable miRNA functional analysis by upregulation of miRNA activity. miRNA inhibitors are small, chemically modified single-stranded RNA molecules designed to specifically bind to and inhibit endogenous miRNA molecules and enable miRNA functional analysis by downregulation of miRNA activity. miR-124 mimics, miR-124 inhibitors and negative control siRNA oligonucleotides were chemosynthesized (Shanghai GenePhama Co., Ltd.). miR-124 mimics were sense: 5′-UAAGGCACGCGGUGAAUGCC-3′, and anti-sense: 5′-CAUUCACCGCGUGCCUUAUU-3′. Negative controls were sense: 5′-UUCUCCGAACGUGUCACGUTT-3′, and anti-sense: 5′-ACGUGACACGUUCGGAGAATT-3′. miR-124 inhibitors (anti-miR-124) were sense: 5′-GGCAUUCACCGCGUGCCUUA-3′. Negative controls of miR-124 inhibitors (Anti-miR-C) were sense: 5′-GUGGAUAUUGUUGCCAUCA-3′. Cells were cultured from 80 to 90% confluence after being seeded into six-well plates, and the oligonucleotide transfection was performed with Lipofectamine 2000 (Invitrogen). Fifty nanomoles per litre of the RNA duplex were used for each transfection. After 48 h of transfection, cells were harvested for further experimentation.

### Treatment with 5-aza-2′-deoxycytidine (DAC)

SKM-1 cells were cultured at a density of 0.5 × 10^6^ cells/mL in 10 mL of RPMI-1640 medium with 10% foetal bovine serum and were maintained at 37 °C in a humid atmosphere containing 5% CO_2_. Cells were treated with 2 and 4 μM DAC (Sigma, Taufkirchen, Germany), respectively. Cells were harvested on days 1, 2, 3, 4 and 5 of DAC treatment.

### RNA isolation and quantitative real-time PCR

Total RNA was extracted from the SKM-1 cells using TRIzol reagent (Invitrogen, San Diego, CA, USA), and small RNAs were isolated using a mirVana miRNA isolation kit (Ambion, Inc., Austin, TX, USA) according to the manufacturer’s instructions. Reverse transcription was performed at 48°C for 15 min. PCR was performed for 40 cycles at 95 °C for 15 s and 60 °C for 60 s. TaqMan microRNA assays were used to quantify the expression levels of mature miR-124 according to the manufacturer’s protocol (Applied Biosystems, San Diego, CA, USA). U6 was used as an internal control. All samples were normalized to internal controls, and the fold changes were calculated according to the relative quantification method (RQ = 2^−ΔΔCT^). The primer sequences were as follows: miRNA Universal R: GTGCAGGGTCCGAGGT; miR-124-F: GCCTAAGGCACGCGGTG; miR-124-RT: GTCGTATCCAGTGCAGGGTCCGAGGTATTCGCACTGGATACGACGGCATT; U6-F: CTCGCTTCGGCAGCACA; U6-R: AACG CT TCACGAATTTGCGT.

### Western blot analysis

For the western blot analyses, total proteins were extracted from the cultured cells and then quantitated using a bicinchoninic acid assay kit (Pierce, Rockford, IL, USA), with bovine serum albumin as a standard. Proteins were fractionated by sodium dodecyl sulphate polyacrylamide gel electrophoresis (SDS-PAGE) transferred to a polyvinylidene fluoride (PVDF) membrane, were blocked in 5% dry milk at room temperature for 1 h and were immunostained overnight at 4 °C using antibodies against CDK6 (Epitomics, Burlingame, CA, USA). An antibody to GAPDH (ProteinTech Group, Chicago, IL) was used as a loading control.

### Cell viability assays

Cells were seeded at 2000 per well in 96-well plates and cultured after transfection. Cell viability was assessed by Cell Counting Kit-8 (CCK-8) assay (Beyotime, Shanghai, China) following the manufacturer’s instructions. Briefly, cells were seeded into a 96-well plate at a concentration of 2 × 10^3^ cells per well. Each well contained 10 μl of CCK-8 in 90 μl of culture medium. The cells were incubated for 2 h at 37 °C, and absorbance was measured at 450 nm. Three independent experiments were performed.

### Cut-off values and statistical analysis

The MDS samples had different levels of DNA methylation. To explore the prognostic significance of the methylation level of each CpG site, we used ROC analysis to obtain the best cut-off values for classifying patients into “high-meth” and “low-meth” groups. To evaluate the predictive value of each gene, such as miR124-1, miR-124-2 or miR-124-3, we first used mean values from all pyrosequenced CpG sites as a measure of methylation of a given gene, as reported in two previous studies [18, 32]. Then, we used ROC analysis to obtain the cut-off values for classifying patients into “high-meth” and “low-meth” groups. When we produced an ROC curve to estimate the sensitivity and specificity of each site or each gene as a prognostic marker for overall survival (OS) or leukaemia-free survival (LFS), we used median OS or median LFS as a criterion. The state variable of patients with a survival time or leukaemia-free survival time of more than the median OS or median LFS was defined as “1”, while for those with less than the median OS or median LFS, it was defined as “0”. The cut-off values of each site and each gene are shown in Tables 1 and 2.

**Table 1:** Overall survival of MDS patients with different miR-124 methylation levels at CpG sites by pyrosequencing

mir-124 genes	Cut-off values (%)	No. of patients		Overall survival (months)		*P*
		Low-meth	High-meth	Low-meth	High-meth	
miR-124-1	4.9	33	23	31.8	8.4	*0.025*
miR-124-1-1	3.5	15	41	32.7	11.6	*0.034*
miR-124-1-2	4.5	15	41	32.7	11.3	*0.048*
miR-124-1-3	3.5	16	40	21.8	11.6	0.209
miR-124-1-4	8.5	44	12	21.8	5.4	*<0.001*
miR-124-2	24.8	27	29	31.8	6.6	*0.004*
miR-124-2-1	11.5	23	33	31.8	11.3	0.125
miR-124-2-2	15	26	30	31.8	6.6	*0.020*
miR-124-2-3	19.5	35	21	21.8	6.6	0.053
miR-124-2-4	16.5	35	21	31.8	6.2	*0.001*
miR-124-2-5	30	24	32	32.7	6.6	*0.001*
miR-124-3	8.6	21	35	32.7	7.6	*0.010*
miR-124-3-1	6.5	15	41	31.8	12.8	0.117
miR-124-3-2	10.5	29	27	31.8	6.6	0.052
miR-124-3-3	4.5	17	39	23.8	7.7	0.306
miR-124-3-4	14.5	19	37	31.8	11.6	0.094
miR-124-3-5	12	37	19	31.8	6.6	*0.004*
miR-124-3-6	4.5	20	36	31.8	7.5	*0.009*
miR-124-3-7	6.5	20	36	31.8	11.3	*0.048*
miR-124-3-8	11.5	19	37	46.5	7.5	*<0.001*
miR-124-3-9	8.5	36	20	31.8	6.6	*0.026*

Italics are statistically significant

**Table 2:** Leukaemia-free survival of MDS patients with different miR-124 methylation levels at CpG sites by pyrosequencing

mir-124 genes	Cut-off values (%)	No. of patients		Leukaemia-free survival (months)		*P*
		Low-meth	High-meth	Low-meth	High-meth	
miR-124-1	7.4	43	13	36.3	12.3	*0.029*
miR-124-1-1	3.5	15	41	36.9	31.9	0.379
miR-124-1-2	4.5	15	41	40.6	25.2	0.148
miR-124-1-3	3.5	16	40	34.9	33.1	0.688
miR-124-1-4	8.5	44	12	36.6	5.5	*0.010*
miR-124-2	24.8	27	29	43.1	18.0	*0.001*
miR-124-2-1	11.5	23	33	40.3	23.5	0.058
miR-124-2-2	15	26	30	42.9	18.3	*0.002*
miR-124-2-3	19.5	35	21	37.2	18.0	0.083
miR-124-2-4	16.5	35	21	38.2	18.4	*0.011*
miR-124-2-5	30	24	32	44.7	18.0	*0.001*
miR-124-3	8.6	21	35	39.5	24.1	0.069
miR-124-3-1	13.5	42	14	35.2	12.5	0.271
miR-124-3-2	10.5	29	27	36.5	24.2	0.178
miR-124-3-3	4.5	17	39	32.1	30.1	0.302
miR-124-3-4	14.5	19	37	34.4	19.2	*0.042*
miR-124-3-5	9.0	31	25	36.9	24.0	0.119
miR-124-3-6	4.5	20	36	39.1	30.4	0.096
miR-124-3-7	7.5	29	27	36.4	30.8	0.279
miR-124-3-8	11.5	19	37	44.3	21.7	*0.005*
miR-124-3-9	8.5	36	20	35.6	21.0	0.254

Italics are statistically significant

Analysis of variance and Student’s *t* tests were used to determine the statistical significance of differences between samples, and the results were expressed as the mean ± s.d. OS was measured from the day of diagnosis until death from any cause or until the last follow-up date. LFS was calculated from the day of diagnosis until progression to acute leukaemia or end of follow-up. The Kaplan–Meier method was used to compare OS or LFS between patients in the low and high methylation groups. A log-rank test was used to estimate differences in survival. The Cox regression model was used for the multivariate survival analysis to identify the significant independent prognostic factors affecting OS or LFS. For all the analyses, a *P* value of <0.05 was considered statistically significant.

## Results

### Overall survival and leukaemia-free survival of MDS patients with different miR-124 methylation levels

We used pyrosequencing to evaluate the methylation levels of upstream regions of the miR-124 genes (miR-124-1, miR-124-2 and miR-124-3). The pyrosequencing assays interrogated four to nine adjacent CpG sites close to the primary (pri)-miR-124 (Fig. 1). To evaluate the prognostic value of each site and each gene, we used ROC analysis, the best cut-off values for which are shown in Tables 1 and 2. We tested the methylation levels of four CpG sites in the miR-124-1 locus, miR-124-1-1, miR-124-1-2, miR-124-1-3 and miR-124-1-4 and found that high miR-124-1-4 predicted worse OS and LFS, and high miR-124-1-1 and miR-124-1-2 indicated only unfavourable OS. For the five CpG sites that we tested in the miR-124-2 locus, we found that high methylation of miR-124-2-2, miR-124-2-4 and miR-124-2-5 predicted shorter OS and higher risk of AML transformation. For miR-124-1 and miR-124-2, high methylation predicted unfavourable OS and LFS. We tested nine CpG sites in the miR-124-3 locus. High miR-124-3 methylation predicted unfavourable survival, and high methylation of miR-124-3-5, miR-124-3-6, miR-124-3-7, miR-124-3-8 and miR-124-3-9 was also correlated with shorter survival. In addition, high miR-124-3-4 and miR-124-3-8 methylation indicated higher risk of AML evolution (Tables 1 and 2). The quantitative DNA methylation of miR-1241-1, miR-124-2 and miR-124-3 in 56 MDS patients and peripheral blood from 10 healthy donors are shown in (Additional file 1: Figure S1).

### Prognostic value of miR-124 methylation and haematological factors

#### Univariate analysis

**Fig. 2:**
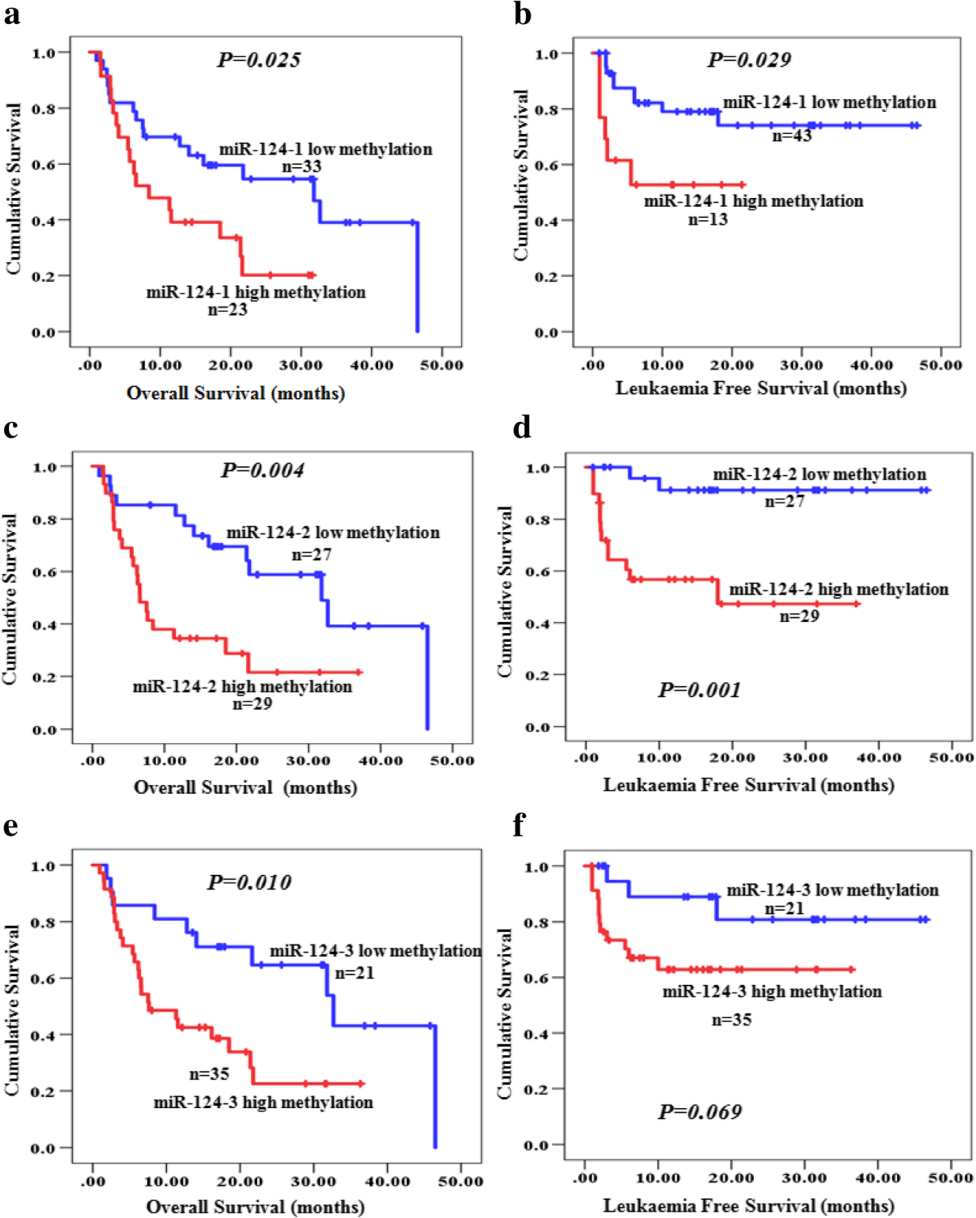
Overall survival (OS) and leukaemia-free survival (LFS) of MDS patients with different levels of miR-124 methylation. **a** OS according to different methylation levels of miR-124-1. **b** LFS according to different methylation levels of miR-124-1. **c** OS according to different methylation levels of miR-124-2 methylation. **d** LFS according to different methylation levels of miR-124-2 methylation. **e** OS according to different methylation levels of miR-124-3 methylation. **f** LFS according to different methylation levels of miR-124-3 methylation

To evaluate the prognostic value of different levels of methylation of miR-124-1, miR-124-2 and miR-124-3, we performed univariate analysis by using the Kaplan–Meier method and log-rank test. Prognostic factors affecting OS and LFS in the univariate analysis are listed in Table 3. Among the laboratory and clinical parameters, the World Health Organization (WHO) classification, marrow blast count, karyotype, International Prognostic Scoring System (IPSS) and mean corpuscular volume were found to be significant prognostic factors for OS in the univariate analysis. For LFS, patients who were older and in the advanced WHO classification stage, with high IPSS scores and high marrow blast counts had higher risk of progression to AML. High methylation levels of miR-124-1 and miR-124-2 were proved to be adverse prognostic factors with shorter OS and LFS in the MDS patients (Fig. 2a–d). For miR-124-3, high methylation predicted shorter OS (*P* = 0.010, Fig. 2e), but it did not significantly impact the LFS (*P* = 0.069, Fig. 2f).

**Table 3:** Prognostic impact of miR-124 methylation levels and haematological factors in patients with MDS

Variables	Overall survival (OS)				Leukaemia-free survival (LFS)		
	No. of	No. of	Univariate	Multivariate	No. of	Univariate	Multivariate
	patients	event	*P*	*P*	event	*P*	*P*
Sex			0.326	–		0.513	–
Male	36	20			9		
Female	20	14			6		
Age			0.103	–		*0.023*	*0.002*
< 60	25	12			3		
≥ 60	31	22			12		
WBC (× 10^9^/L)			0.932	–		0.313	–
≥ 4	13	8			2		
< 4	43	26			13		
Hb(g/dL)			0.058	–		0.050	–
≥ 10	9	4			0		
< 10	47	30			15		
PLT (× 10^9^/L)			0.469	–		0.702	–
≥ 100	18	9			4		
< 100	38	25			11		
MCV(fL)			*0.014*	0.111		0.861	–
≥ 100	28	13			8		
< 100	28	21			7		
WHO classification			*<0.001*	*0.016*		*0.003*	0.944
SLD/RS-SLD/ MDS-U/5q-	10	5			0		
MLD/RS-MLD	10	2			0		
EB-1/EB-2	36	27			15		
BM blast (%)			*<0.001*	*0.003*		*<0.001*	0.453
< 5	20	7			0		
5–10	15	10			3		
11–19	21	17			12		
Cytopenias			0.391	–		0.19	–
0/1	11	5			1		
2/3	45	29			14		
Karyotype			*0.005*	*<0.001*		0.626	–
Good	34	18			9		
Intermediate	7	5			2		
Poor	14	11			4		
IPSS			*0.002*	*0.007*		*0.004*	0.122
Low	4	1			0		
Int-1	24	12			2		
Int-2	19	14			10		
High	8	7			3		
miR-124-1 methylation			*0.025*	0.810		*0.029*	0.125
Low	33^a^,43^b^	17			9		
High	23 ^a^,13^b^	17			6		
miR-124-2 methylation			*0.004*	0.312		*0.001*	0.307
Low	27	13			2		
High	29	21			13		
miR-124-3 methylation			*0.010*	*0.028*		0.069	-
Low	21	10			3		
High	35	24			12		

Different cut-off values of methylation levels were used to predict OS and LFS (Tables 1 and 2); therefore, ^a^means the number of patients with low or high methylation levels to predict OS, and ^b^means the number of patients with low or high methylation levels to predict LFS

Italics are statistically significant

#### Multivariate analysis

To assess the prognostic value of factors found to be significant in the univariate analysis, we performed a multivariate Cox regression analysis of their effect on OS and LFS. As shown in Table 3, in addition to the WHO classification, marrow blast, karyotype and IPSS, high methylation of miR-124-3 was an independent factor of OS. The median survival of patients with high miR-124-3 methylation was 7.6 months, significantly shorter than patients with low methylation (median survival of 32.7 months, *P* = 0.010; Fig. 2e). According to the multivariate analysis, age was the only independent factor that predicted worse LFS (Table 3).

### Correlation between different levels of miR-124-3 methylation and clinical parameters

Based on the results of multivariate analysis, which showed that high miR-124-3 methylation was the independent risk factor of OS, we analysed the correlation between different levels of miR-124-3 methylation and clinical factors. As shown in Table 4, high methylation of miR-124-3 was independent of sex, white blood cell count, haemoglobin level, platelet count, mean corpuscular volume and karyotype and cytopenias at initial diagnosis. However, patients with the advanced WHO classification or high marrow blast count had a significantly higher frequency of miR-124-3 high methylation. There is a slight trend that patients with intermediate or high IPSS risk had higher incidence of miR-124-3 high methylation, it did not reach the statistical significance (Table 4).

**Table 4:** Correlation between different levels of miR-124-3 methylation and clinical parameters

Variables	No.	miR-124-3 methylation		*P*
		Low-meth (n%)	High-meth (*n*%)	
Sex				0.150
Male	36	11(30.6)	25(69.4)	
Female	20	10(50.0)	10(50.0)	
Age				*0.015*
< 60	25	5(20.0)	20(80.0)	
≥ 60	31	16(51.6)	15(48.4)	
WBC (× 10^9^/L)				1.000
≥ 4	13	5(38.5)	8(61.5)	
< 4	43	16(37.2)	27(62.8)	
Hb(g/dL)				0.715
≥ 10	9	4(44.4)	5(55.6)	
< 10	47	17(36.2)	30(63.8)	
PLT (× 10^9^/L)				0.184
≥ 100	18	9(50.0)	9(50.0)	
< 100	38	12(31.6)	26(68.4)	
MCV(fL)				0.783
≥ 100	28	11(39.3)	17(60.7)	
< 100	28	10(35.7)	18(64.3)	
WHO classification				*0.006*
SLD/RS-SLD/ MDS-U/5q-	10	7(70.0)	3(30.0)	
MLD/RS-MLD	10	6(60.0)	4(40.0)	
EB-1/EB-2	36	8(22.2)	28(77.8)	
BM blast (%)				*0.006*
< 5	20	13(65.0)	7(35.0)	
5–10	15	3(20.0)	12(80.0)	
11–19	21	5(23.8)	16(76.2)	
Cytopenias				0.298
0/1	11	6(54.5)	5(45.5)	
2/3	45	11(33.3)	30(66.7)	
Karyotype				0.888
Good	34	13(38.2)	2 (61.8)	
Intermediate	7	2(28.6)	5(71.4)	
Poor	14	5(35.7)	9(64.3)	
IPSS				0.358
Low	4	3(75.0)	1(25.0)	
Int-1	24	9(37.5)	15(62.5)	
Int-2	19	6(31.6)	13(68.4)	
High	8	2(25.0)	6(75.0)	

Italics are statistically significant

### miR-124 suppresses cell proliferation in the SKM-1 cell line

**Fig. 3:**
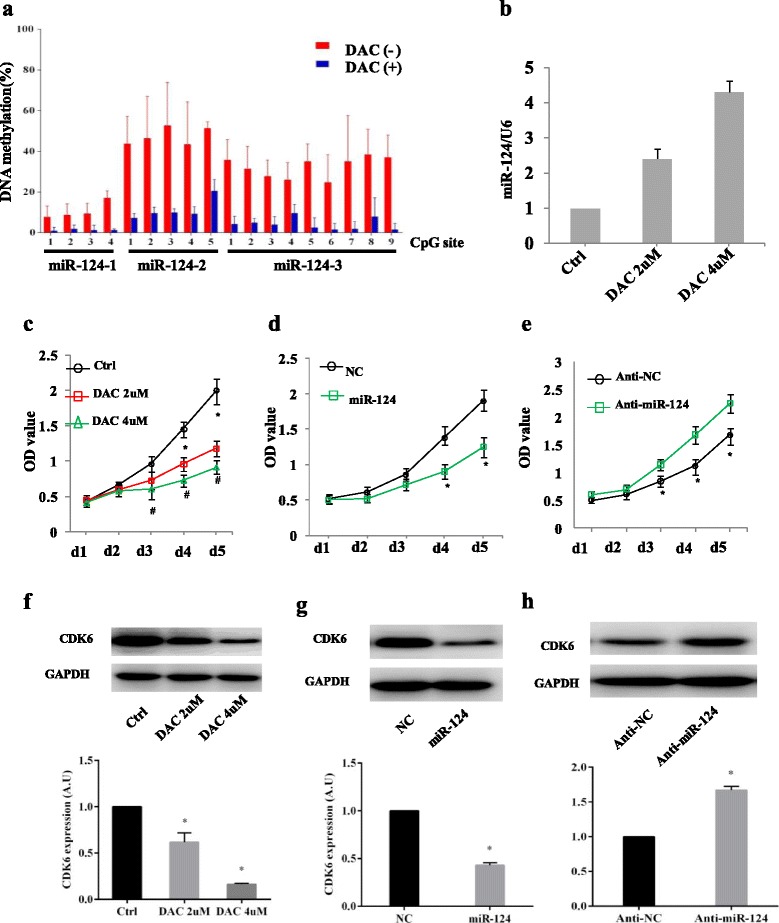
miR-124 suppresses cells proliferation and downregulates the expression of CDK6 in SKM-1 cell line. **a** Methylation status of miR-124 inSKM-1 cells before and after 4 μM DAC treatment for 48 h. **b** SKM-1 cells were treated with 2 and 4 μM DAC for 48 h, and miR-124 expression was determined by real-time PCR and normalized to U6. The results are presented as the means ± s.d. of values obtained in three independent experiments. Statistical significance was calculated using the Student’s *t* test. * *P* < 0.05. **c** The effect of miR-124 on cell proliferation was measured by CCK-8 assay. Increased expression of miR-124 after DAC treatment inhibited cells proliferation. **d** The effect of miR-124 overexpression on cell proliferation. miR-124 means SKM-1 cells transfected with miR-124 mimics, and NC means negative control of SKM-1 cells transfected with miR-124 mimics. **e** The effect of miR-124 suppression on cell proliferation. Anti-miR-124 means the cells transfected with miR-124 inhibitors, and anti-NC means the negative control of cells transfected with miR-124 inhibitors. **f** Western blot and a quantitative analysis of bands intensity of CDK6 in SKM-1 cells treated with 2 and 4 μM DAC for 48 h. GAPDH served as the internal control. **g** The effect of miR-124 overexpression on endogenous CDK6 expression was measured by western blot and a quantitative analysis of band intensity. GAPDH served as an internal control. miR-124 means SKM-1 cells transfected with miR-124 mimics, and NC means negative control of SKM-1 cells transfected with miR-124 mimics. **h** The effect of miR-124 suppression on endogenous CDK6 expression was measured by western blot and a quantitative analysis of band intensity. GAPDH served as an internal control. Anti-miR-124 means the cells transfected with miR-124 inhibitors, and anti-NC means the negative control of cells transfected with miR-124 inhibitors

We first examined the methylation status of miR-124-1, miR-124-2 and miR-124-3 in the SKM-1 cell line, and the results showed that methylation levels significantly decreased after DAC treatment (Fig. 3a). Then we tested the expression of miR-124 in SKM-1 cell line and found that after DAC treatment, there was a significant increase in the expression of miR-124 (Fig. 3b). We also evaluated the effects of miR-124 on cell proliferation in MDS. In vitro cell proliferation assays revealed that increased expression of miR-124 induced by DAC significantly inhibited MDS cell proliferation (Fig. 3c). Furthermore, miR-124 mimics or miR-124 inhibitors were transfected to SKM-1 cells. After transfection, miR-124 expression was determined by real-time PCR and normalized to U6 (Additional file 1: Figure S2). We found that in miR-124 mimics transfected SKM-1 cells, cell proliferation was significantly suppressed (Fig. 3d), while a miR-124 inhibitor, which significantly decreased endogenous miR-124 expression, promoted cell proliferation (Fig. 3e). Taken together, these data indicate a proliferation–inhibitory role of miR-124 in MDS.

### miR-124 targets CDK6 expression in the SKM-1 cell line

To investigate the potential mechanism of how miR-124 regulates its target genes, we focused on the gene encoding CDK6 because it has been confirmed as a target in previous studies [19, 23, 28]. Using western blot analyses, we observed that the SKM-1 cells, which had low miR-124expression levels, strongly expressed CDK6, while the cells treated with the demethylating agent DAC showed CDK6 downregulation (Fig. 3f). To further confirm the targeting of CDK6 by miR-124, SKM-1 cells were transfected with miR-124 mimic, miR-124 inhibitor or the respective controls. The result showed that CDK6 was decreased in cells transfected with miR-124 mimics, but increased in cells with the miR-124 inhibitor (Fig. 3g, h). These data indicate that CDK6 was targeted by miR-124, and that epigenetic silencing of miR-124 in SKM-1 cells led to CDK6 upregulation.

## Discussion

In this study, we found that miR-124 genes (miR-124-1, miR-124-2 and miR-124-3) were highly methylated in MDS patients, and increased methylation levels of several CpG sites are associated with shorter OS or LFS. In addition, we found that miR-124-3 was associated with adverse clinical parameters, including high blast count and advanced WHO classification. These data suggested that miR-124 genes, which are, at least, mediated through an epigenetic mechanism, might be involved in the pathogenesis of MDS. This view was further supported by our findings that high methylation of miR-124-3 was significantly associated with decreased OS in both the univariate and in multivariate analyses. The clinical relevance of miR-124 methylation as an independent prognosticator has been investigated in several previous studies. In renal cell cancer, miR-124-3 high methylation was shown to be associated with disease recurrence [33]. In pancreatic cancer, high methylation of miR-124-2 and miR-124-3 was correlated with decreased survival time [18] and, in another study, the methylation level of miR-124-3 was shown be to be associated with increased risk of developing gastric cancer [34]. In haematological malignancies, Castoro et al. first reported the methylation status of miR-124-1 and miR-124-3 in MDS and AML, and identified miR-124-1 methylation as a prognostic marker for OS [21]. Roman-Gomez reported a shortened disease-free survival for patients with ALL using a multi-miRNA loci approach that included miR-124-3 [35]. Subsequently, methylation of the miR-124-3 locus was identified as an independent prognosticator for both disease-free survival and OS in ALL [23]. In addition, Castoro et al. demonstrated that dynamic changes in miR-124-1 and miR-124-3methylation may predict the clinical response after DAC therapy in MDS patients [21]. MiR-124-2 has been found to be methylated in both healthy lymphocytes and various types of cancer [17, 18, 23, 35]. Although shorter OS and LFS in MDS patients with high miR-124-2 and miR-124-3 methylation were found in the univariate analysis, these correlations were not significant in the multivariate analysis.

Recent studies have investigated the biological function of miR-124 in various cancers, and increasing evidence revealed that miR-124 can regulate the expression of a variety of genes pivotal for tumorigenesis and progression [18, 24–27, 36, 37]. Wang et al. reported that miR-124 was epigenetically silenced in pancreatic cancer and inhibited cell proliferation and metastasis by regulating Rac1 [18]. Zheng et al. showed that miR-124 was frequently downregulated in hepatocellular carcinoma and played an important role in the invasion and migration by regulating ROCK2 and EZH2 [36]. Han et al. found that miR-124 was downregulated in breast cancer and that the ectopic expression of miR-124 could suppress invasion and metastatic ability by directly targeting CD151 [37]. In haematological malignancies, a functional study by Agirre et al. revealed that downregulation of miR-124 by methylation induced the upregulation of *CDK6* and phosphorylation of retinoblastoma, which contributed to the abnormal proliferation of ALL cells both in vitro and in vivo [23]. In AML, miR-124-1 and miR-124-3 were found to be methylated, and upregulation induced by DAC treatment of epigenetically silenced miR-124 can lead to downregulation of its two targets, CDK6 and CCAAT/Enhancer Binding Protein ɑ(C/EBPɑ) [22]. Jeong et al. showed that miR-124 acted as a tumour-suppressive miRNA in B-cell lymphomas by reducing MYC and BCL2 expression through the targeting of p65 [38]. Castoro et al. reported the methylation status of miR-124-1 and miR-124-3 in MDS patients and suggested that the re-expression of miR-124 was a good marker of response to DAC [21]. Another recent study also demonstrated that high-risk MDS or AML patients who responded to epigenetic treatment showed significant induction of miR-124 and inhibition of CDK4 and CDK6 expression [39]. However, the role of miR-124 in cell proliferation and the regulation of its target have not been clearly elucidated in vitro. In the present study, we tested the effect of miR-124 on SKM-1 cells. Our results showed that miR-124 re-expression after DAC treatment could suppress MDS cell growth. We also showed that synthetic miR-124 mimics inhibited cell growth, which suggests its role as a tumour suppressor in MDS. Furthermore, we validated CDK6 as a target of miR-124 by western blot analysis in vitro. Our findings suggest that post-transcriptional of CDK6 by miR-124 is a vital mechanism underlying cell proliferation, and miR-124 may serve as a potential treatment target for regulating CDK6 to inhibit cell proliferation in MDS.

There are several limitations to our study. First, the patients cohort of 56 cases included in the current study were diagnosed between June 2003 and April 2007, and during this period, demethylating agents were not approved by the China Food and Drug Administration. Therefore, no patients in our study received a demethylating agent such as decitabine as standard therapy. Second, we tested the methylation levels of the miR-124 loci only at the time of initial diagnosis and not after treatment; therefore, whether miR-124 methylation levels could predict responsiveness to demethylating agents has not been established. Third, the present study is retrospective and had a small sample size. Finally, because of the lack of frozen cells or mRNAs, miR-124 functional study was performed only in the SKM-1 cell line, with no further studies being performed in clinical patient samples. Several previous studies reported the silencing of the expression of miR-124 regulated by methylation in MDS, AML and ALL [20, 21, 23]. Our present study confirmed that miR-124-3 high methylation was an independent risk factor for OS in MDS patients. All three upstream regions of the miR-124 genes (miR-124-1, miR-124-2 and miR-124-3) express miR-124, and miR-124 negatively regulates its target mRNA transcripts, typically through base pairing with a section in the 3′-untranslated region (3′-UTR). Therefore, whether miR-124 expression at diagnosis was correlated with patient prognosis needs to be verified in future study. In the DAC era, dynamic examination of miR-124 methylation, miR-124 expression and its targeted genes in MDS patients both before and after DAC treatment may be crucial to resolve this issue.

## Conclusions

In conclusion, we found that miR-124 methylation was a common molecular event in MDS, and that high methylation of miR-124-3 was associated with poor overall survival in MDS. The functional study revealed that miR-124 targeted CDK6 and inhibited cell proliferation in vitro, which suggests its role as a tumour suppressor.

## Additional file

Additional file 1: Figure S1. Quantitative DNA methylation of miR-1241-1, miR-124-2 and miR-124-3 in 56 MDS patients and peripheral blood from 10 healthy donors. Each row represents a sample, and each column represents a single CG site. Colour coding reflects the degree of methylation with yellow being 100% and blue being 0%. Figure S2**.** After transfection with miR-124 mimics (**a**) and miR-124 inhibitors (**b**) in SKM-1 cells, miR-124 expression was determined by real-time PCR and normalized to U6. (PDF 55 kb)

## Abbreviations

Hb: Haemoglobin
IPSS: International Prognostic Scoring System
Karyotype: Good: normal, 5q- sole, 20q- sole, −Y sole
Intermediate: Not good or poor
Poor: Complex aberration or chromosome 7 abnormalities
MCV: Mean corpuscular volume
MDS: Myelodysplastic syndrome
PLT: Platelets
WBC: White blood cell
WHO classification: World Health Organization classification
