# Supplementary material for: Pyrosequencing quantified methylation level of miR-124 predicts shorter survival for patients with myelodysplastic syndrome
Source: Clin Epigenetics. 2017 Aug 30;9:91. doi: 10.1186/s13148-017-0388-5 (PMC5577794; doi:10.1186/s13148-017-0388-5)

Additional file

Additional figure legends

Additional Figure 1. Quantitative DNA methylation of miR-1241-1 ,miR-124-2 and miR-124-3 in 56 MDS patients and peripheral blood from 10 healthy donors . Each row represents a sample and each column represents a single CG site. Color coding reflects the degree of methylation with yellow being 100% and blue being 0%

Additional Figure 2. After transfection with miR-124 mimics (a) and miR-124 inhibitors(b) in SKM-1 cells, miR-124 expression was determined by real-time PCR and normalized to U6.

Additional Figure 1. Quantitative DNA methylation of miR-1241-1, miR-124-2 and miR-124-3 in 56 MDS patients and peripheral blood from 10 healthy donors.

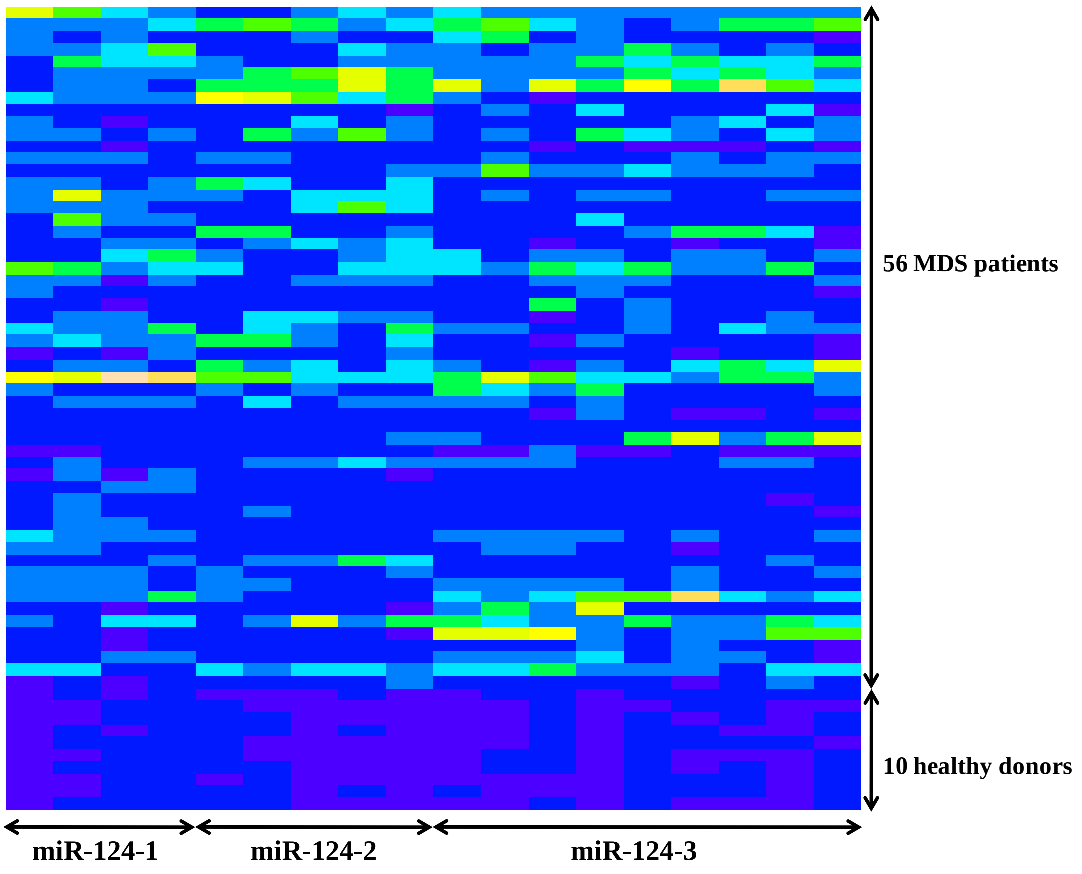

Additional Figure 2. miR-124 expression in SKM-1 cells after transfection with miR-124 mimics and inhibitors.

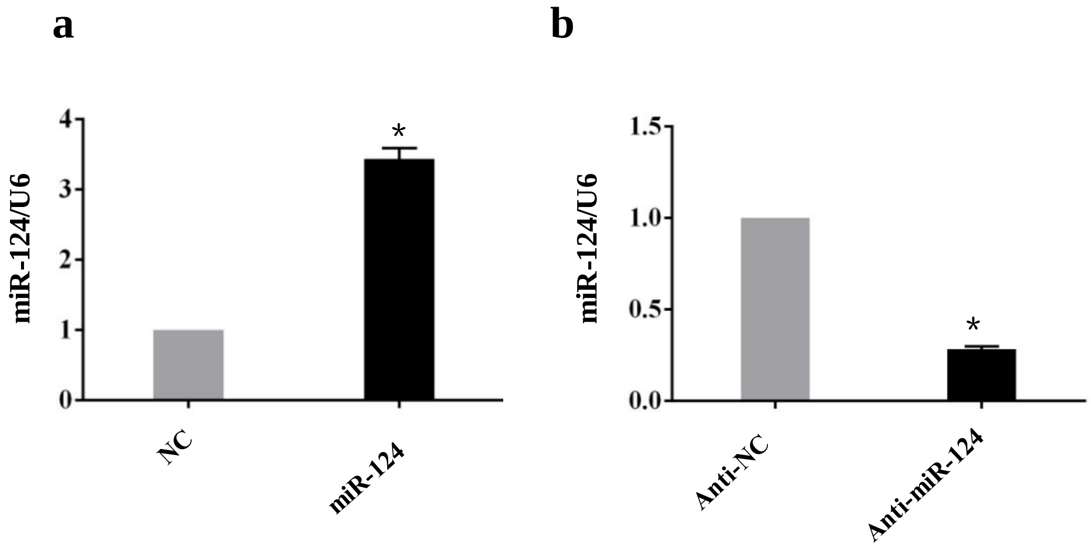

Supplement: Additional file 1: Figure S1. — Quantitative DNA methylation of miR-1241-1, miR-124-2 and miR-124-3 in 56 MDS patients and peripheral blood from 10 healthy donors. Each row represents a sample, and each column represents a single CG site. Colour coding reflects the degree of methylation with yellow being 100% and blue being 0%. Figure S2. After transfection with miR-124 mimics (a) and miR-124 inhibitors (b) in SKM-1 cells, miR-124 expression was determined by real-time PCR and normalized to U6. (PDF 55 kb) [file 13148_2017_388_MOESM1_ESM.pdf]
